# Medication regimen complexity in cancer patients: an overlooked issue for healthcare team

**DOI:** 10.1007/s00520-025-09476-9

**Published:** 2025-05-01

**Authors:** Sude Ayca Cifci, Elif Aras Atik, Ömer Dizdar, Aygin Bayraktar-Ekincioglu

**Affiliations:** 1 Faculty of Pharmacy, Department of Clinical Pharmacy, Hacettepe University, Sihhiye, Ankara, 06100 Turkey; 2 Institute of Oncology, Department of Medical Oncology, Hacettepe University, Ankara, Turkey

**Keywords:** Adherence, Medication regimen complexity, Medication burden, Unplanned hospitalization

## Abstract

**Purpose:**

The primary aim of this study was to assess the medication regimen complexity in patients with cancer and the change in complexity from admission to discharge. The secondary aim of the study was to explore the impact of medication complexity on the length of hospital stay, patients’ perceived medication burden, adherence, and unplanned hospitalizations after discharge.

**Methods:**

This study was prospectively conducted in medical oncology clinics of a tertiary care hospital. Patients over 18 years of age and diagnosed with solid tumors and deemed non-palliative were included. A clinical pharmacist assessed the patient’s medication regimen complexity using the medication regimen complexity index (MRCI) upon admission, at 48 h of hospitalization, and at discharge. The clinical pharmacist also participated in the patient education provided by the multidisciplinary team at discharge. Patients’ adherence was assessed by the medication adherence reporting scale (MARS) at admission and at the first outpatient visit after the discharge. The perceived burden of drug treatment by the patients was assessed using a questionnaire named the medication complexity questionnaire from patient perspective (MCQPP) that was designed by the researchers. The questionnaire was administered at the first follow-up visit at the outpatient clinic after the discharge. Unplanned hospitalizations of the patients within 30 days after discharge were also examined.

**Results:**

A total of 147 patients were enrolled. The median (IQR) MRCI score at discharge was 15.0 (11.0–22.0), which was significantly higher than the MRCI score at admission, which was 11.0 (7.0–15.0) (*p* < 0.001). As a result of the patient education provided at discharge by the multidisciplinary team, a statistically significant increase was observed in the treatment adherence of the patients. The median (IQR) MARS score assessed at admission was 19 (15–23), while it increased to 20 (16–23) at the follow-up outpatient visit (*p* = 0.003). Complexity was found to be higher in patients who had more negative perceptions about their medication, in patients who reported that their daily life was significantly affected by their medication, and in those who perceived more significant burden when complex instructions for medication use were evident (*p* < 0.001). The median MRCI scores at discharge were higher in patients with unplanned hospitalizations compared to those without hospitalization (*p* < 0.001).

**Conclusions:**

In conclusion, integrating the MRCI into oncology practices, along with multidisciplinary care that includes clinical pharmacists, may enable the identification of high-risk patients, the individualization of drug treatment, and the optimization of the treatment process.

**Supplementary Information:**

The online version contains supplementary material available at 10.1007/s00520-025-09476-9.

## Introduction

Polypharmacy is a commonly observed health issue among cancer patients and challenges the treatment and care of patients. Complications related to cancer and its treatment, advancing age, and comorbidities lead to an increase in the number of medications used by patients and complexity in drug therapies [1, 2]. Although the number of medications affects the complexity of a medication regimen, other factors also contribute, including dose form, dosing frequency, and additional directions for medication use (e.g., need to take with food). The medication regimen complexity index (MRCI) is a tool developed to objectively quantify medication regimen complexity [3].

Medication regimen complexity is considered to be an independent risk factor for adverse health outcomes [4]. High medication complexity has been associated with various drug-related problems (DRP) (e.g., nonadherence [5], adverse drug reactions [6], potentially inappropriate medications [7]), hospital readmission [8], prolonged hospital stays [9], hospital discharge to an aged care facility [10], decreased quality of life [11], increased health costs [12], or even high mortality [13]. Moreover, medication complexity creates a medication-related burden for patients, affecting patients’ treatment satisfaction, psychological well-being, social functioning and quality of life, as well as leading to nonadherence and other negative clinical outcomes [14, 15]. Assessment of medication related burden perceived by the patients and its impact on patients’ behavior regarding drug usage may help to provide individualized care services in accordance with the patient’s daily routine and to increase the quality of health services delivered [14, 15].

The complexity of treatment in cancer patients and the presence of chronic diseases cause DRP to be frequently seen in this group of patients [16]. Integration of a clinical pharmacist in oncology healthcare settings has been reported to reduce adverse drug reactions, untreated indications, potentially inappropriate medications, and drug-drug interactions, resulting in optimal pharmaceutical care and increased patient safety [17–19]. Comprehensive pharmaceutical care services provided by clinical pharmacists are important in increasing patients’ adherence and beliefs on drug therapy, and therefore improving therapeutic outcomes [19, 20]. Pharmacist-physician collaboration can simplify complex drug therapies and reduce adverse health outcomes [21].Therefore, the primary objective of this study was to evaluate the medication regimen complexity in patients with cancer, and to ascertain the extent of any change in complexity from the time of admission to that of discharge. The secondary objective of the study was to investigate the impact of medication complexity on the length of hospitalization, patients’ perceived medication burden, adherence, and unplanned hospitalizations following discharge.

## Method

The study was prospectively conducted between June 2022 and April 2023 in a tertiary care hospital and approved by the University Ethics Committee (No: GO-22–399). Patients aged ≥ 18 years diagnosed with solid tumors, regularly taking > 1 medication, scheduled for hospitalization for 48 h or longer due to the assessments of the patients within 48 h of their hospitalization, the necessary consultations, and the initiation of medical treatments for the reason of hospitalization, and who were able to communicate were considered eligible for participation in the study. Patients diagnosed with hematological malignancies, and those who transferred from another ward and/or intensive care unit to the ward were excluded from the study, as it would not be feasible for the clinical pharmacist participating in the study to provide daily follow-ups. Since clinical pharmacy services were not provided in the palliative care ward in the study setting and the patients at the terminal stage who were hospitalized in the palliative wards considered not able to manage their medication by themselves, this study was undertaken in medical oncology wards, in which terminal stage cancer patients were excluded.

**Fig. 1:**
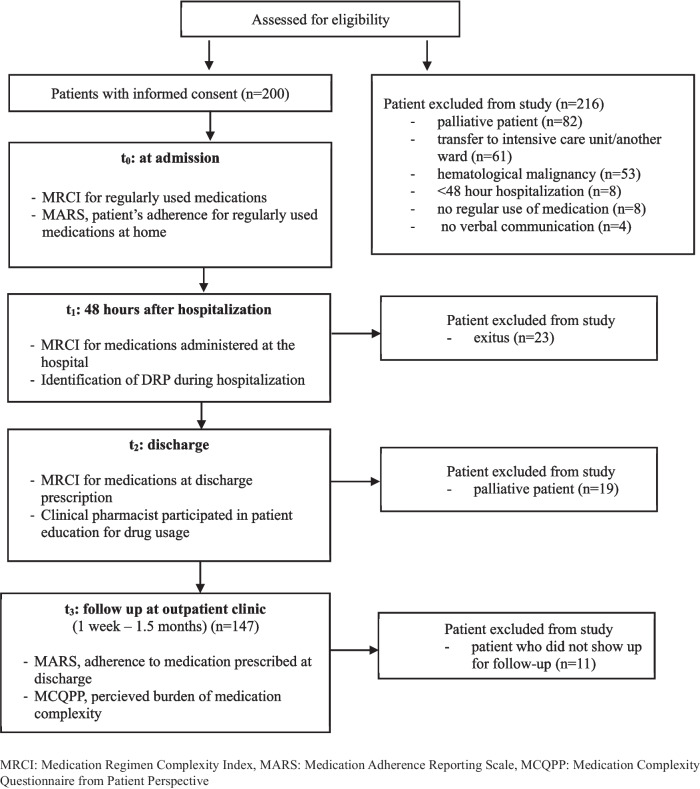
Flow diagram of the study

The sample size was calculated with 90% power and a 5% margin of error based on a previous study [22] evaluating the change in the total MRCI score at hospitalization to the total MRCI score at discharge and considering the patient density in the ward where the study was conducted, and it was determined that a minimum of 137 patients should be included in the study. The flow diagram of the study is shown in Fig. 1. Patient demographics, medications, and relevant clinical information were collected from patient medical files, hospital information systems, and from the patients themselves.

For the purpose of the study, a clinical pharmacist was integrated into the oncology wards to collaborate with physicians and nurses. The clinical pharmacist provided follow-up care for patients from the time of admission until the time of discharge, and then at the first outpatient clinic visit. A clinical pharmacist evaluated patients’ adherence, assessed the patients’ medication regimen complexity from admission until discharge, and identified any DRPs that occurred during hospitalization. The clinical pharmacist also participated in patient education on medication usage given by a physician at discharge. Unplanned hospitalizations within a 30-day period after discharge were also monitored.

Medication regimen complexity was assessed by using the MRCI tool, which comprises 65 items that evaluate the dosage forms (section A), dosing frequencies (section B), and additional instructions (section C) for each medication administered [3]. The medication complexity was evaluated at the time of admission (for regularly used medications), 48 h after the admission (for medications used during hospitalization) and at the discharge (for discharge prescription). The time points for the assessments were selected to reflect the complexity of the patients’ medications at home, during hospitalization (once all treatments for the reason of hospitalization have been initiated), and at discharge (where the patient continues to take medications). The Turkish validity and reliability of the scale was previously reported [23].

Patients’ adherence was assessed by the medication adherence reporting scale (MARS) at admission and at the first outpatient visit after the discharge (1 week to 1.5 months) [24]. The scale consists of 5 items on medication-taking behaviors (forget to take; change the dose; stop taking for a while; decide to skip a dose; take less than prescribed) and assessed the behavior on a Likert scale (5 = never, 4 = rarely, 3 = sometimes, 2 = often, 1 = always). The total score is calculated by summing the scores obtained for each item and varies between 5 and 25. An increase in the total score indicates adherence, and a decrease in the total score indicates non-adherence [24, 25]. The Turkish validity and reliability of the scale was previously reported [23, 25].

The clinical pharmacist identified DRPs and made recommendations to physicians, nurses, or patients in order to solve the problems.

The burden of drug treatment as perceived by patients was assessed using a questionnaire named as medication complexity questionnaire from patient perspective (MCQPP) that was designed by the researchers. The questionnaire was administered at the first follow-up visit at the outpatient clinic after the discharge. The comprehensibility of the questionnaire was evaluated by a pilot study of 10 outpatients’ responses, and no revision was required. The questionnaire investigates patients’ perceived burden of drug treatment by 6 sub-headings with a total of 20 questions on 5-point Likert scale: (a) “perceived impact” (3 questions evaluate the effects of medication and side effects on patients’ quality of life); (b) “practical difficulties” (6 questions assess medication adherence and patients’ perceived medication complexity); (c) “general concerns” (3 questions assess patients’ awareness of drug interactions and their knowledge about drug therapies); (d) “financial burden” (1 question assesses patients’ economic concerns about drug therapies); (e) “interference with daily life” (4 questions evaluates the effects of medication and side effects on patients’ daily routines and social lives); and (f) “communication with health personnel” (3 questions asses patients’ satisfaction with the counseling of doctors and pharmacists regarding their treatment).

### Statistical analysis

For statistical analysis, the IBM SPSS version 26.0 program was used. The data were analyzed after normality analysis; Spearman’s correlation coefficient was used for the relationship between numerical data, Mann-Whitney *U*, and Kruskal-Wallis analysis of variance were used to compare categorical data, and the Wilcoxon test was used for the difference between numerical data. The Friedman test was used to examine the change in the number of medications and MRCI scores. Generalized estimating equations (GEE) of the regression analysis was used to evaluate the effect of the number of medications and adherence scores on the MRCI score over time. Statistical significance was considered as *p* < 0.05. Since th MCQPP was not normally distributed, the results of the questionnaire were presented as a 3-point Likert scale (never-rarely; usually; always).

## Results

A total of 147 patients were included in the study (Fig. 1). The median age (interquartile range, IQR) was 63 (54–70) years, and the majority of the patients (*n* = 135, 92.5%) had comorbid conditions (Table 1). A total of 722 drugs were used in 147 patients (4.09 drugs/patient), and the most commonly used agents were gastrointestinal medications (*n* = 77), renin-angiotensin system blocking drugs (*n* = 48), antithrombotic medications (*n* = 45), and analgesics (*n* = 44).

**Table 1:** Characteristics of the patients (*n* = 147)

	*n* (%)
Gender	
Male	83 (56.5)
Age (median, IQR)	63 (54–70)
Cancer diagnosis	
Lung cancer	33 (22.4)
Colon cancer	19 (12.9)
Breast cancer	17 (11.6)
Stomach cancer	12 (8.2)
Pancreatic cancer	12 (8.2)
Liver and intrahepatic canal cancers	9 (6.1)
Ovarian cancer	8 (5.4)
Rectum cancer	5 (3.4)
Others^a^	32 (21.8)
Chronic diseases	
Cardiovascular diseases	91 (61.9)
Type 2 diabetes	43 (29.3)
Psychiatric diseases	22 (15.0)
Gastrointestinal diseases	21 (14.3)
Benign prostatic hyperplasia	21 (14.3)
Diseases of the thyroid gland	16 (10.9)
Chronic lower respiratory diseases	14 (9.5)
Dyslipidemia	11 (7.5)
Others^b^	46 (31.3)
Number of chronic diseases	
0	12 (8.2)
1	37 (25.2)
2	45 (30.6)
≥ 3	53 (36.1)
Number of medications used regularly	
< 5	74 (50.3)
5–9	67 (45.6)
≥ 10	6 (4.1)
Length of hospital stay (day) (median, IQR)	13 (7–19)
Reasons for hospitalization	
Complications of anticancer drugs	98 (66.7)
Receiving anticancer drugs	30 (20.4)
Supportive care (for pain and nutrition)	7 (4.8)
Interventional procedures	7 (4.8)
Pleural effusion	5 (3.4)
ECOG (median, IQR)	2 (1–2)
Cancer diagnosis, year (median, IQR)	12 (3–16)
Treatment protocol (active treatment) (***n* = **126 patients)	
Chemotherapy	81 (64.3)
Chemotherapy + targeted therapy	17 (13.5)
Targeted therapy	9 (7.1)
Chemotherapy + immunotherapy	6 (4.8)
Immunotherapy	6 (4.8)
Hormonal therapy	4 (3.2)
Hormonal therapy + targeted therapy	2 (1.6)
Hormonal therapy + immunotherapy	1 (0.8)

*IQR* interquartile range, *ICD* International Classification of Diseases, *ECOG* Eastern Cooperative Oncology Group

Others^a^: Renal cell carcinoma (*n* = 4), esophageal cancer (*n* = 4), prostate cancer (*n* = 3), cervical cancer (*n* = 3), skin malignant neoplasm (*n* = 2), hypopharyngeal cancer (*n* = 2), bladder cancer (*n* = 2), neuroendocrine tumor (*n* = 2), testicular cancer (*n* = 2), brain malignant neoplasm (*n* = 1), endometrial cancer (*n* = 1), laryngeal cancer (*n* = 1), mesenchymal tumor (*n* = 1), mesothelioma (*n* = 1), thyroid cancer (*n* = 1), thymus malignant neoplasm (*n* = 1), synovial sarcoma (*n* = 1)

Others^b^: Chronic renal failure (*n* = 8), epilepsy (*n* = 4), chronic liver disease (*n* = 3), hepatitis B (*n* = 3), cerebrovascular disease (*n* = 3), osteoporosis (*n* = 2), anemia (*n* = 2), glaucoma (*n* = 1), dry eye syndrome (*n* = 1), Meniere’s (*n* = 1), gout (*n* = 1), Behçet’s (*n* = 1)

In terms of cancer and its treatment, the most common types of cancer were lung (22.4%), colon (12.9%) and breast cancer (11.6%). Among patients, 57 (38.8%) have received radiotherapy (alone/in combination), 136 (92.5%) have received anticancer drug treatment, and 126 (85.7%) currently receive anticancer drug treatment. More than half of the patients (*n* = 98, 66.7%) received supportive treatment for complications of cancer treatment.

When MRCI scores were calculated at 3 time points (admission, 48 h after admission, discharge) and the number of medications was compared, significant changes were found between the 3 time points (*p* < 0.001). As a result of treatment changes during hospitalization, the MRCI score from admission to discharge increased in 105 (71.4%) patients, decreased in 32 (21.8%) patients, and remained unchanged in 10 (6.8%) patients. The median (IQR) MRCI score at 48 h after admission was 22.5 (15.0–28.5), which was higher than the MRCI scores both at admission and at discharge (Table 2).

**Table 2:** Number of medications and MRCI scores in patients during the study

Median (IQR)					
	At admission (*t*_0_)	During hospitalization (*t*_1_)	At discharge (*t*_2_)	*p*	Pairwise comparisons^b^
Number of medications	4 (3–7)	8 (6–10)	6 (5–8)	**< 0.001**^**a**^	***t***_**0**_**–*t***_**1**_**: *p* < 0.05** ***t***_**1**_**–*t***_**2**_**: *p* < 0.05** **t**_**0**_**–t**_**2**_**: *p* < 0.05**
MRCI total score	11 (7–15)	22.5 (15–28.5)	15 (11–22)	**< 0.001**^**a**^	***t***_**0**_**–*t***_**1**_**: *p* < 0.05** ***t***_**1**_**–*t***_**2**_**: *p* < 0.05** ***t***_**0**_**–*t***_**2**_**: *p* < 0.05**
Dosage forms (section-A) score	3 (1–4)	8 (5–10)	4 (1–6)	**< 0.001**^**a**^	***t***_**0**_**–*t***_**1**_**: *p* < 0.05** ***t***_**1**_**–*t***_**2**_**: *p* < 0.05** ***t***_**0**_**–*t***_**2**_**: *p* < 0.05**
Dosing frequency (section-B) score	6.5 (4–9)	12.5 (8.5–16.5)	8 (6–12)	**< 0.001**^**a**^	***t***_**0**_**–*t***_**1**_**: *p* < 0.05** ***t***_**1**_**–*t***_**2**_**: *p* < 0.05** ***t***_**0**_**–*t***_**2**_**: *p* < 0.05**
Additional directions (section-C) score	1 (1–3)	2 (1–3)	2 (1–4)	**< 0.001**^**a**^	*t*_0_–*t*_1_: *p* = 0.568 *t*_1_–*t*_2_: *p* = 0.133 ***t***_**0**_**–*t***_**2**_**: *p* < 0.003**

*IQR* interquartile range

^a^Changes in the number of medications and MRCI scores over time were analyzed by the Friedman test

^b^The Bonferroni test was performed to find out which groups were responsible for the difference

According to the GEE analysis, a higher number of medications was found to be associated with higher MRCI scores when the effect of the number of medications was adjusted for the effect of the MRCI score at all three assessment periods (*p* < 0.001). Once the number of medications was considered, the MRCI score calculated at admission was lower, and the MRCI score calculated at 48 hours after the admission was found to be higher, compared to the MRCI score at discharge (*p* < 0.001) (Table 3).

**Table 3:** The effect of the number of medications on the MRCI score

Parameter estimation				
Parameter	Adjusted coefficient	Standard error	95% Confidence intervals	*p*
Time (Reference: MRCI – *t*_2_)				
MRCI – *t*_0_	− 1.057	0.2923	(− 1.630–− 0.484)	**< 0.001**
MRCI –* t*_1_	2.283	0.4395	(1.421–3.144)	**< 0.001**
Number of medications	2.484	0.1163	(2.256–2.712)	**< 0.001**

*t*_*0*_ at admission, *t*_*1*_ 48 h after hospitalization, *t*_*2*_ at discharge

By the provision of patient education at discharge, the MARS score increased in 53.7% (*n* = 79), decreased in 23.1% (*n* = 34) and remained the same in 23.1% (*n* = 34) of the patients. The median (IQR) MARS score assessed at admission was 19 (15–23), whereas it was 20 (16–23) at the follow-up outpatient visit (*p* = 0.003). The effect size of time on the MARS score was found to be 25% (small effect size).

Between the scores of MARS and MRCI, a significant but weak negative correlation was found at admission (*p* = 0.001; *r*_*s*_ = − 0.263), and a significant moderate negative correlation was found between discharge MRCI and MARS score at the follow-up outpatient clinic visit (*p* = 0.001; *r*_*s*_ = − 0.433). According to the GEE analysis performed to evaluate the effect of the change in MARS score on the MRCI total score according to time, the patients with higher MARS scores had lower MRCI scores (*p* < 0.001) (Table 4).

**Table 4:** The effect of observed change in the MARS score on the MRCI score

Parameter estimation				
Parameter	Adjusted coefficient	Standard error	95% Confidence intervals	*p*
Time (Reference: MRCI – *t*_2_)				
MRCI – *t*_0_	− 5.093	0.4653	(− 6.005–− 4.181)	**< 0.001**
Change in MARS score	− 0.712	0.1202	(− 0.948–− 0.477)	**< 0.001**

t_0_ at admission, *t*_*2*_ at discharge

During hospitalization, 149 DRPs were identified by the clinical pharmacist (median number of DRP/patient: 1). The identified problems were related with “treatment safety (*n* = 79),” “treatment effectiveness (*n* = 47),” and “other reasons (*n* = 23),” of which the main causes were “drug dose of a single active ingredient too high (21.5%),” “no or incomplete drug treatment in spite of existing indication (11.4%),” “dosage regimen too frequent (10.1%),” and “inappropriate drug according to guidelines/formulary (8.7%).” One hundred and forty-one recommendations (94.6%) for physicians and 3 (2%) for patients were suggested to address these DRPs, while 5 (3.4%) did not require any specific recommendations. The interventions included dosage changes (*n* = 63), stop drug (*n* = 23), start drug (*n* = 18), instructions for changed drug use (*n* = 15), drug change (*n* = 12), formulation change (*n* = 8), and monitoring of drug (*n* = 5). Of the 144 interventions made by the clinical pharmacist, 120 (83.3%) were accepted and fully implemented by the physician and the patient. Of the interventions, 57 were related to the treatment of comorbidities, 44 to the treatment of infections, 30 to supportive treatment, and 13 to cancer treatment. A positively moderate correlation was found between the number of DRP/patient and MRCI scores at admission (*p* < 0.001, *r*_*s*_ = 0.263), during hospitalization (*p* < 0.001, *r*_*s*_ = 0.242), and at discharge (*p* < 0.001, *r*_*s*_ = 0.223).

Among the patients, 29.3% (*n* = 43) had an unplanned hospitalization within 30 days after the discharge. The MRCI [median (IQR)] scores at discharge were higher in patients with unplanned hospitalizations [24.00 (18.00–28.50)] compared to patients without hospitalization [12.25 (9.50–16.75)] (*p* < 0.001). Despite the examination of confounders (such as comorbidity burden, age, and treatment) in predicting unplanned hospitalizations through a univariate analysis, no significant relationship was identified between the variables of gender, age, Charlson comorbidity index, and the MRCI score at discharge. No significant correlation was found between the total MRCI score at admission and the length of hospital stay (*p* = 0.074, *r*_*s*_ = 0.148).

The results of the MCQPP questionnaire revealed that patients who express negative perceptions about their treatment, who have difficulties in complying with their treatment, who feel more burdened in cases with specific instructions, who need more information about medications, who indicate that medication side effects affect their daily and social lives, and who feel they could not get adequate information from doctors and pharmacists, exhibited higher MRCI scores (*p* < 0.001) (Suppl. 1).

When the relationship between the patient’s perceived medication complexity and adherence was evaluated, it was found that patients who had a more positive perception of their treatment (*p* = 0.007), able to tolerate side effects (*p* = 0.010), reported having an improved quality of life as a result of the treatment (*p* = 0.006), and stated that they received adequate information from pharmacists (*p* < 0.001) have an increase in the MARS score, while patients who had difficulty in complying with treatment (*p* = 0.002) and encountered problems in accessing their medications (*p* = 0.030) have a decrease in the MARS score (Suppl. 2).

## Discussion

Previous studies have largely been conducted in geriatric patients in order to evaluate medication complexity in hospitals, where chronic diseases and polypharmacy are frequently observed [10, 26, 27]. Therefore, this is the first study that investigated medication regimen complexity in hospitalized cancer patients. The medications used for comorbidities and supportive therapies, in addition to anticancer treatment, led to the consideration of cancer patients as high-risk individuals in terms of adverse patient outcomes such as unplanned hospitalization, decreased quality of life, and increased prevalence of DRPs, including non-adherence.

In studies evaluating medication regimen complexity, it is seen that the MRCI score varies between 9 and 45 [22, 27–30]. Although the MRCI score in this study was lower than the studies in the literature, it may be explained by the fact that the patients in the study were younger and the number of chronic diseases per patient was lower compared to other studies. Cancer is a remarkably complex disease, and intensive anticancer treatment, long outpatient infusions, and additional visits for supportive care further complicate the management of cancer. Taken together, the need for prolonged and increasingly complex treatment can be viewed as unique stressors in cancer [31]. A high MRCI score does not always indicate “very complex” treatment. Therefore, when assessing the complexity, the clinical condition of the patient, the differences in administration route of the drugs, and the duration of drug usage (such as chronic treatment) should also be taken into consideration.

Similar to the results of hospitalized patients, an increase in patients’ MRCI scores was observed with hospitalization in this study [22, 26]. An increase in the number of medications and changes in drug formulations (e.g., switching oral to transdermal opioids) as a result of patients having new diagnoses (e.g., initiation of oral suspension for the management of mucositis) or modification of treatment (e.g., prescribing low molecular weight heparin for the risk of venous thromboembolism) during hospitalization can increase the medication regimen complexity [4]. The increase in MRCI score seen under the control of physicians and clinical pharmacists during hospitalization is not an uncontrolled increase, but is due to medication regimen optimization.

Unplanned hospitalization was one of the most common health care outcomes studied in association with the MRCI score. Because the MRCI score significantly changes during the course of the hospital stay, the discharge MRCI score can reasonably be used to predict the risk of a post-discharge readmission. In the study, the MRCI score assessed at the discharge was associated with unplanned hospitalization within 30 days, which supports that the MRCI score may predict a marker of hospitalizations and can be used to identify patients at risk [8, 30]. It was previously demonstrated that opioids, anticoagulants, non-steroidal anti-inflammatory drugs, cardiovascular system drugs, sedatives, anxiolytics, and antipsychotics had been associated with recurrent unplanned hospitalizations [9]. Similarly, the intensive use of these groups of drugs, especially analgesics and cardiovascular system drugs, in the treatment of cancer patients participating in the study may be a reason for unplanned hospitalization within a 30-day period after discharge.

The studies investigated the association between medication complexity and the duration of hospitalization, encouraging the use of the MRCI tool in daily clinical practice [32, 33]. Given the fact that the health condition of cancer patients is generally critical, the use of the MRCI assessment tool in practice will be valuable to predict patients at risk [9].

Improving patient adherence is crucial to reducing hospital readmissions. Physician-pharmacist collaboration on the modification of drug treatment and the provision of information for patients about drugs may reduce the perceived medication regimen complexity and increase patient adherence. In an oncology ward, patient education on drug treatment provided by a clinical pharmacist has positive effects on medication adherence [20, 34–36]. In this study, the clinical pharmacist was involved in the multidisciplinary healthcare team for the provision of education. The MARS score at follow-up outpatient visit was found to be higher than the score at the time of hospitalization. It is thought that the increase in adherence, despite the increase in the MRCI score, is due to the effect of the medication education given to the patients. The fact that cancer patients have frequent outpatient clinic visits compared to other chronic patients, and that these outpatient clinic visits involve various specialties, may result in high disease awareness. This is thought to be a factor that increases patients’ adherence. Over time, patients may establish a routine in the use of medication or lose the habitual use of medication. Therefore, the measurement of MARS at different times may result in an increase or decrease in adherence, depending on individual characteristics.

Although there have been studies [14, 15, 37] which evaluated the medication treatment burden perceived by patients with chronic diseases, none of these studies included cancer patients. Drug-related factors such as the requirement of the patients to use different dosage forms (patches, suspensions) or additional instructions to use (changing fentanyl patches every 48 h) can make the treatment more complex and ultimately result in an increase in the patient’s perceived burden for cancer treatment.

An increase in the perception of drug-related burden decreases patients’ adherence to drug treatment [37]. The observed increase in adherence in patients who responded “usually and always” to the statement of “I can easily adapt to the times when I have to take my medication” in the MCQPP questionnaire, which may indicate adherence, may reflect the positive effect of patient education provided by the multidisciplinary team. Understanding the perception related to treatment burden, as well as medication regimen complexity in patients, and designing therapeutic care plans appropriate for patients’ lifestyles may create opportunities for healthcare professionals to provide individualized patient care [14]. Development and routine use of validated tools for evaluation of medication complexity and patients’ perceived burden on treatment will help to improve the quality of service provided in patient care processes. Such services can be designed to include the MRCI scale, serving as a patient prioritization tool that predicts outcomes such as unplanned hospitalization, increased length of hospital stay, and drug interactions, in particular for patients who are at high risk and have a burden on health costs.

The medication burden may also arise from DRPs. Although there were not many DRPs observed for anticancer drugs in this study, drugs used in the treatment of infection and supportive care resulted in a more frequent occurrence of DRPs. Since the clinical pharmacist made recommendations to address DRPs and physicians accepted 83.3% of these recommendations, the majority of the DRPs were resolved. This may have indirectly reduced the impact of treatment burden.

The study has limitations; subgroup analyses could not be performed for cancer type and treatment protocols due to limited numbers of patients included. The study was conducted in a single center; however, it is one of the major/well-known cancer care centers in the country, which may reflect the tertiary care practices for oncology. Since this study was conducted in the medical oncology wards and did not include patients in a palliative care ward, the patient empowerment in early palliative care is still needed to be explored in future studies. As no previous study evaluated the medication-related complexity in cancer patients, the study results cannot be compared and interpreted any further. In addition, patients’ adherence to drug therapy was evaluated in a very short period of time after discharge, which may not reflect the real-world situation. While many validated quality of life scales generally evaluate the effect of the disease on the patients’ daily living, since there is no validated scale that evaluates the impact of perceived burden of medication complexity, researchers assessed this situation by developing the MCQPP. Due to time constraints, the MCQPP could not be validated during the study process. The intention is to conduct the requisite studies for the validation of the MCQPP in order to create a standardized scale that will facilitate evaluation from the patient perspective. It may lead to the development of such instruments that comprehensively evaluate the burden related to disease, medication, and treatment processes in cancer patients.

Future studies should be designed to investigate the burden of medication complexity in the long term, and to surrogate the impact of clinical pharmacy services in the management of treatment complexity by inclusion of a control group in patients with cancer.

## Conclusions

In order to ensure that patients receive the best possible care, it is essential that new methods of identifying those at risk of poor outcomes are developed. Assessing medication regimen complexity in patients may enable the multidisciplinary healthcare team to prioritize patients’ care issues, optimizing health outcomes through medication therapy management in order to predict the risk of readmission. In the future, there should be a great focus on longer term, larger scale studies, including a control group, with the aim of simplifying complex medication therapies by assessing the impact of the clinical pharmacist.

Consequently, considering the complexity of medication in the monitoring of cancer patients and evaluating the complexity of medication from the patients’ perspective may contribute to the improvement of multidisciplinary health services.

## Supplementary Information

Below is the link to the electronic supplementary material.

Supplementary file 1 (PDF 115 KB)

Supplementary file 2 (PDF 117 KB)

## Data Availability

No datasets were generated or analysed during the current study.
