# Supplementary material for: Medication regimen complexity in cancer patients: an overlooked issue for healthcare team
Source: Support Care Cancer. 2025 May 1;33(5):440. doi: 10.1007/s00520-025-09476-9 (PMC12045823; doi:10.1007/s00520-025-09476-9)
Supplement: Supplementary file 1 — (PDF 115 KB) [file 520_2025_9476_MOESM1_ESM.pdf]

Title: Medication regimen complexity in cancer patients: an overlooked issue for healthcare team

Journal Name: Supportive Care in Cancer

Author:

Sude Ayca Cifci[1], Elif Aras Atik[1], Ömer Dizdar[2], Aygin Bayraktar-Ekincioglu[1]

[1] Hacettepe University, Faculty of Pharmacy, Department of Clinical Pharmacy, Ankara-Turkiye

[2] Hacettepe University, Institute of Oncology, Department of Medical Oncology, Ankara-Turkiye

Corresponding Author:

Aygin Bayraktar-Ekincioglu

Hacettepe University Faculty of Pharmacy, Department of Clinical Pharmacy, 06100 Sihhiye - Ankara, Turkey

Email: aygin@hacettepe.edu.tr

Supplement 1 :The relationship between MCQPP responses and MRCI score

	Discharge MRCI score median (IQR)	p-value
**Perceived impact**		
1. I think my medication is effective.		
Never/Rarely	24.0 (16.5 – 27.5)	**<0.001**
Usually	15.0 (11.25 – 19.25)	
Always	10.0 (6.5 – 15.0)	
2. I can say that my quality of life has improved thanks to my medications.		
Never/Rarely	23.0 (16.5 – 27.5)	**<0.001**
Usually	15.0 (11.0 – 19.0)	
Always	8.5 (6.5 – 12.5)	
3. I can ignore the side effects I experience because I benefit from my medication.		
Never/Rarely	22.75 (15.0 – 26.5)	**<0.001**
Usually	15.0 (11.5 – 18.0)	
Always	8.5 (5.5 – 17.0)	
**Practical difficulties**		
4. I find it difficult to get my medication.s prescribed by the doctor(s).		
Never/Rarely	15.0 (11.0 – 20.0)	0.149
Usually	24.0 (10.0 – 28.5)	
Always	10.0 (4.0 – 27.0)	
5. I can easily adapt to the times when I have to take my medications.		
Never/Rarely	23.0 (13.5 – 27.5)	**<0.001**
Usually	15.0 (11.5 – 20.5)	
Always	11.0 (6.5 – 15.5)	
6. I keep a record of the medication.s I have and can make a routine of taking my medications.		
Never/Rarely	22.75 (13.5 – 27.0)	**<0.001**
Usually	16.25 (12.0 – 24.0)	
Always	11.0 (6.5 – 15.0)	
7. I think that having special instructions to follow for my medications complicates my medication use.		
Never/Rarely	7.25 (5.25 – 11.0)	**<0.001**
Usually	15.0 (11.0 – 18.0)	
Always	17.75 (12.0 – 26.0)	
8. I feel that taking my medications more than once a day is a burden on me.		
Never/Rarely	11.0 (5.5 – 15.0)	**<0.001**
Usually	13.25 (10.0 – 17.0)	

Always	17.75 (13.0 – 25.75)	
9. I feel that taking different formulations of medications during the day is a burden on me.		
Never/Rarely	9.0 (6.5 – 15.0)	**<0.001**
Usually	14.0 (11.0 – 16.5)	
Always	19.0 (13.5 – 26.5)	
**General concerns**		
10. I am worried about my medications interacting with each other.		
Never/Rarely	12.5 (6.75 – 21.25)	0.077
Usually	15.0 (11.0 – 20.5)	
Always	17.5 (11.0 – 22.75)	
11. I am worried that my medications may interact with the food I eat.		
Never/Rarely	15.0 (11.0 – 22.75)	0.968
Usually	14.75 (11.0 – 21.0)	
Always	15.0 (10.0 – 19.5)	
12. I need more information about my medications.		
Never/Rarely	11.0 (6.5 – 15.0)	**<0.001**
Usually	16.0 (12.0 – 23.0)	
Always	17.5 (11.75 – 25.75)	
**Financial burden**		
13. I worry about paying for my medications (within the scope of Social Security Institution reimbursement).		
Never/Rarely	15.0 (11.0 – 20.0)	0.079
Usually	17.0 (12.0 – 25.75)	
Always	27.0 (27.0 – 27.0)	
**Interference in daily life**		
14. My medication causes problems in my daily tasks that require physical and mental strength (housework, work, etc.).		
Never/Rarely	9.0 (6.5 – 14.0)	**<0.001**
Usually	15.0 (12.0 – 19.0)	
Always	24.0 (18.25 – 28.0)	
15. I cannot spare time for social activities (walking, exercise, etc.) and hobbies because of my medication.		
Never/Rarely	11.0 (8.0 – 15.0)	**<0.001**
Usually	16.25 (12.0 – 21.25)	
Always	23.75 (16.5 – 27.5)	
16. I think that my medications have an impact on my basic needs (sleep patterns, eating habits, sexual life).		
Never/Rarely	6.5 (4.0 – 9.0)	**<0.001**
Usually	13.75 (10.0 – 16.5)	
Always	20.0 (14.0 – 27.0)	
17. Side effects affect my daily life (home, work, sleep, food, etc.).		
Never/Rarely	4.0 (4.0 – 6.5)	**<0.001**
Usually	14.0 (10.0 – 16.5)	
Always	19.25 (13.0 – 26.5)	
**Communication with healthcare staff**		
18. I trust my doctor's knowledge and choices as I know he/she will choose the most appropriate medication for me.		
Never/Rarely	18.0 (12.0 – 26.0)	**0.018**
Usually	16.5 (12.0 – 24.5)	
Always	14.25 (9.0 – 18.25)	
19. When I have a problem with my medication, my doctor can give me enough information about my medication.		
Never/Rarely	19.0 (10.0 – 26.0)	**0.001**
Usually	16.5 (12.0 – 25.5)	
Always	14.0 (9.0 – 17.0)	
20. When I have a problem with my medicines, my pharmacist can give me enough information about my medicines.		
Never/Rarely	20.25 (15.0 – 27.5)	**<0.001**
Usually	15.5 (11.5 – 22.75)	
Always	11.0 (8.0 – 16.5)	
