# Supplementary material for: Medication regimen complexity in cancer patients: an overlooked issue for healthcare team
Source: Support Care Cancer. 2025 May 1;33(5):440. doi: 10.1007/s00520-025-09476-9 (PMC12045823; doi:10.1007/s00520-025-09476-9)
Supplement: Supplementary file 2 — (PDF 117 KB) [file 520_2025_9476_MOESM2_ESM.pdf]

Title: Medication regimen complexity in cancer patients: an overlooked issue for healthcare team

Journal Name: Supportive Care in Cancer

Author:

Sude Ayca Cifci[1], Elif Aras Atik[1], Ömer Dizdar[2], Aygin Bayraktar-Ekincioglu[1]

[1] Hacettepe University, Faculty of Pharmacy, Department of Clinical Pharmacy, Ankara-Turkiye

[2] Hacettepe University, Institute of Oncology, Department of Medical Oncology, Ankara-Turkiye

Corresponding Author:

Aygin Bayraktar-Ekincioglu

Hacettepe University Faculty of Pharmacy, Department of Clinical Pharmacy, 06100 Sihhiye - Ankara, Turkey

Email: aygin@hacettepe.edu.tr

Supplement 2: The relationship between MCQPP responses and change in MARS score

	**Change in MARS score median (IQR)**	**p-value**
**Perceived impact**		
1. I think my medication is effective.		
Never/Rarely	-4.55 (-15.38 – 0)	**0.007**
Usually	7.68 (0 – 17.65)	
Always	4.17 (0 – 9.52)	
2. I can say that my quality of life has improved thanks to my medications.		
Never/Rarely	0 (-15.38 – 7.14)	**0.006**
Usually	7.29 (0 – 17.65)	
Always	4.26 (0 – 9.52)	
3. I can ignore the side effects I experience because I benefit from my medication.		
Never/Rarely	-2.27 (-15.38 – 10.0)	**0.010**
Usually	5.26 (0 – 21.05)	
Always	4.17 (0 – 10.53)	
**Practical difficulties**		
4. I find it difficult to get my medication.s prescribed by the doctor(s).		
Never/Rarely	4.55 (0 – 14.29)	**0.030**
Usually	-6.0 (-16.67 – 0)	
Always	5.26 (0 – 11.11)	
5. I can easily adapt to the times when I have to take my medications.		
Never/Rarely	0 (-15.79 – 0)	**0.002**
Usually	7.68 (0 – 17.65)	
Always	4.17 (0 – 9.31)	
6. I keep a record of the medication.s I have and can make a routine of taking my medications.		
Never/Rarely	0 (-16.67 – 10.0)	0.156
Usually	4.65 (-9.52 – 16.67)	
Always	4.35 (0 – 9.52)	
7. I think that having special instructions to follow for my medications complicates my medication use.		
Never/Rarely	6.52 (4.17 – 9.52)	0.440
Usually	4.55 (0 – 11.76)	
Always	0 (-11.11 – 15.79)	
8. I feel that taking my medications more than once a day is a burden on me.		
Never/Rarely	4.17 (0 – 9.52)	0.168
Usually	7.29 (0 – 16.67)	

Always	0 (-11.11 – 14.29)	
9. I feel that taking different formulations of medications during the day is a burden on me.		
Never/Rarely	4.45 (0 – 9.52)	0.542
Usually	7.29 (0 – 10.53)	
Always	0 (-11.76 – 17.65)	
**General concerns**		
10. I am worried about my medications interacting with each other.		
Never/Rarely	4.95 (-4.35 – 10.56)	0.796
Usually	0 (0 – 13.64)	
Always	4.90 (0 – 15.79)	
11. I am worried that my medications may interact with the food I eat.		
Never/Rarely	2.08 (-9.11 – 10.82)	0.149
Usually	5.21 (0 – 23.08)	
Always	0 (0 – 9.09)	
12. I need more information about my medications.		
Never/Rarely	5.95 (0 – 10.53)	0.545
Usually	4.35 (-8.70 – 14.29)	
Always	0 (-11.15 – 16.25)	
**Financial burden**		
13. I worry about paying for my medications (within the scope of Social Security Institution reimbursement).		
Never/Rarely	4.45 (0 – 13.64)	0.410
Usually	0 (-11.56 – 10.56)	
Always	11.1 (11.1 – 11.1)	
**Interference in daily life**		
14. My medication causes problems in my daily tasks that require physical and mental strength (housework, work, etc.).		
Never/Rarely	4.26 (0 – 9.52)	0.763
Usually	4.55 (0 – 13.64)	
Always	0 (-13.39 – 24.04)	
15. I cannot spare time for social activities (walking, exercise, etc.) and hobbies because of my medication.		
Never/Rarely	4.35 (0 – 9.52)	0.339
Usually	5.56 (0 – 16.72)	
Always	0 (-14.29 – 23.08)	
16. I think that my medications have an impact on my basic needs (sleep patterns, eating habits, sexual life).		
Never/Rarely	4.35 (0 – 9.31)	0.292
Usually	5.56 (0 – 12.92)	
Always	0 (-11.76 – 15.79)	
17. Side effects affect my daily life (home, work, sleep, food, etc.).		
Never/Rarely	8.70 (0 – 9.52)	0.478
Usually	4.76 (0 – 11.11)	
Always	0 (-11.44 – 16.72)	
**Communication with healthcare staff**		
18. I trust my doctor's knowledge and choices as I know he/she will choose the most appropriate medication for me.		
Never/Rarely	-4.55 (-20.0 – 0)	0.056
Usually	4.35 (-9.52 – 14.29)	
Always	4.76 (0 – 13.48)	
19. When I have a problem with my medication, my doctor can give me enough information about my medication.		
Never/Rarely	0 (-10.0 – 4.55)	0.093
Usually	4.35 (-9.52 – 20.0)	
Always	5.26 (0 – 13.33)	
20. When I have a problem with my medicines, my pharmacist can give me enough information about my medicines.		
Never/Rarely	-12.77 (-17.39 – 0)	**<0.001**
Usually	4.45 (-2.27 – 14.29)	
Always	8.70 (0 – 15.79)	
